# Dynamics of Biofilm Formation and the Interaction between *Candida albicans* and Methicillin-Susceptible (MSSA) and -Resistant *Staphylococcus aureus* (MRSA)

**DOI:** 10.1371/journal.pone.0123206

**Published:** 2015-04-13

**Authors:** Chaiene Evelin Zago, Sónia Silva, Paula Volpato Sanitá, Paula Aboud Barbugli, Carla Maria Improta Dias, Virgínia Barreto Lordello, Carlos Eduardo Vergani

**Affiliations:** 1 Department of Dental Materials and Prosthodontics, Araraquara Dental School, UNESP—Univ Estadual Paulista. Araraquara, São Paulo, Brazil; 2 IBB-Institute for Biotechnology and Bioengineering, Center of Biological Engineering, University of Minho, Braga, Portugal; University Hospital of the Albert-Ludwigs-University Freiburg, GERMANY

## Abstract

Polymicrobial biofilms are an understudied and a clinically relevant problem. This study evaluates the interaction between *C*. *albicans*, and methicillin- susceptible (MSSA) and resistant (MRSA) *S*. *aureus* growing in single- and dual-species biofilms. Single and dual species adhesion (90 min) and biofilms (12, 24, and 48 h) were evaluated by complementary methods: counting colony-forming units (CFU mL^-1^), XTT-reduction, and crystal violet staining (CV). The secretion of hydrolytic enzymes by the 48 h biofilms was also evaluated using fluorimetric kits. Scanning electron microscopy (SEM) was used to assess biofilm structure. The results from quantification assays were compared using two-way ANOVAs with Tukey post-hoc tests, while data from enzymatic activities were analyzed by one-way Welch-ANOVA followed by Games-Howell post hoc test (α = 0.05). *C*. *albicans*, MSSA and MRSA were able to adhere and to form biofilm in both single or mixed cultures. In general, all microorganisms in both growth conditions showed a gradual increase in the number of cells and metabolic activity over time, reaching peak values between 12 h and 48 h (ρ<0.05). *C*. *albicans* single- and dual-biofilms had significantly higher total biomass values (ρ<0.05) than single biofilms of bacteria. Except for single MRSA biofilms, all microorganisms in both growth conditions secreted proteinase and phospholipase-C. SEM images revealed extensive adherence of bacteria to hyphal elements of *C*. *albicans*. *C*. *albicans*, MSSA, and MRSA can co-exist in biofilms without antagonism and in an apparent synergistic effect, with bacteria cells preferentially associated to *C*. *albicans* hyphal forms.

## Introduction

Biofilms are described as a microbial population attached to a substrate, surrounded by a self-derived extracellular matrix. This mode of life presents important clinical repercussions since it is estimated that over half of all hospital infections are originated from these microbial communities [1]. The relevance of this is that biofilm cells are phenotypically distinct from their ‘free-living’ or planktonic forms, exhibiting elevated resistance to host defenses and higher tolerance to antimicrobial agents [2]. Besides the colonization of the oral mucosal surfaces, biofilms can develop on abiotic substrates, including the internal surfaces of dentures [2,3]. Therefore, biofilms act as reservoirs of pathogenic microorganisms [3–5] favoring the dissemination of infection to other body sites [6,7]. Synergistic, mutualistic, and antagonistic interactions that occur between microorganisms contribute to the development of polymicrobial biofilm communities [8,9]. Currently, the *Staphylococcus aureus* and *Candida* species are ranked among the top three bloodstream pathogens causing severe morbidity and mortality in hospitalized patients [4,10]. Besides the fact that *Candida albicans* and *S*. *aureus* are responsible for a substantial number of infections independently, there is increasing evidence that they can be commonly associated as co-infector microorganisms [5,11–14]. The clinical outcome of these mixed bacterial-fungal interactions is that the infections can correlate with increased frequency or severity of disease [11,15,16]. Infections due to polymicrobial biofilms have also been related to significantly higher mortality rates (70%) when compared to infections caused by a single species of microorganism (23%) [17].

*C*. *albicans* is a commensal colonizer of oral mucous membranes that can become an opportunistic pathogen and is considered the most prevalent [3] and virulent [18] of the *Candida* species. Despite its high virulence as a single pathogen, it has been estimated that about 27% of patients with candidemia had polymicrobial blood cultures, with *S*. *aureus* as the third most common organism isolated in conjunction with *C*. *albicans* [19]. *S*. *aureus* methicilin-resistant (MRSA) has also been recognized as one of the most virulent human pathogens, especially due to its ability to develop resistance against several drug therapies [20,21] and to form biofilms [14,22,23]. In fact, approximately 52% of nosocomial infections in patients in intensive care units are due to MRSA [24], which is responsible for severe infections, such as aspiration pneumonia [25,26].

There is increasing evidence in the literature regarding the importance of polymicrobial infections and some studies have focused on the interactions between the eukaryotic pathogen *C*. *albicans* and the prokaryotic pathogen *S*. *aureus* [5,11–14]. At the moment, it is known that the synergy between these pathogens in a biofilm results in a stronger biofilm formation with increased antimicrobial tolerance [5,12]. In addition, higher mortality rates in mice co-infected with sublethal levels of these microorganisms have been observed [11]. Despite of these studies, some gaps in the virulence attributes of these microorganisms in biofilms still remain to be investigated. In polymicrobial communities, the effect of these specific virulence factors may be influential and perhaps potentiate the pathogenicity of these biofilms. Among these virulence factors, the secretion of specific degradative enzymes can be highlighted as one of the most significant, since they are involved in colonization and infection processes [27,28]. To the author’s knowledge, the quantification of some hydrolytic enzymes in polymicrobial biofilms has not yet been conducted. Despite the fact that *C*. *albicans* and *S*. *aureus* are often co-isolated in cases of biofilm associated infections [29], literature on the interactions between these pathogens is limited and should be further explored in a more comprehensive way. Thus, the aim of the present study was to investigate the dynamic interaction between *C*. *albicans* and methicillin-susceptible (MSSA) and-resistant *S*. *aureus* (MRSA) in terms of characterization of the viable cells, metabolic activity, and total biomass of biofilms. In addition, the quantification of the extracellular hydrolytic enzymes aspartyl proteinase (SAP) and phospholipase C (PL-C) in these biofilms using a highly sensitive fluorimetric method was evaluated.

## Material and Methods

### Strains and growth conditions

Three reference strains were used in this study. *Candida albicans* wild-type strain from the Spain Collection (SC5314), a human clinical isolate, was selected based on its high ability to grow in the invasive hyphal form [30,31]. Two reference *Staphylococcus aureus* from the American Type Culture Collection Methicillin-susceptible *Staphylococcus aureus* (MSSA—ATCC 25923) and methicillin-resistant *S*. *aureus* (MRSA—ATCC 33591) strains were selected because they are capable of biofilm formation in vitro [32,33]. In addition, these strains were also used in other investigations where the interaction of *Candida*-*Staphylococcus* biofilms was evaluated [5,12–14].

*C*. *albicans* was maintained in Yeast Peptone Glucose medium (YEPD: 1% yeast extract, 2% Bacto peptone and 2% D-glucose, 2% agar) and frozen at -70°C until use. The microorganism was subcultured onto Sabouraud Dextrose Agar plates (SDA—Acumedia Manufactures Inc., Baltimore, MD, USA) [34] supplemented with chloramphenicol (0.05 g L^-1^) and incubated at 37°C for 24–48 h to generate the *C*. *albicans* yeast used for all the experiments.

Bacteria strains were maintained in Tryptic Soy Broth medium (TSB—Acumedia Manufactures Inc., Baltimore, MD, USA) [33] and frozen at -70°C until use. The microorganisms were subcultured onto Mannitol Salt Agar plates (Acumedia Manufactures Inc., Baltimore, MD, USA) [33] and incubated at 37°C for 24–48 h to generate the bacteria cells used for the experiments.

To prepare the yeast and bacteria inocula, a loop full of the agar stock cultures was transferred to 5 mL of Yeast Nitrogen Base broth (YNB—Difco, Becton Dickinson Sparks, MD, USA) supplemented with 100 mM glucose and TSB, respectively, and incubated at 37°C overnight in an orbital shaker (75 rpm). Cells of the resultant cultures were harvested and washed twice with phosphate-buffered saline solution (PBS, pH 7.2) at 5,000 x g for 5 min (rotor model A462). Washed microorganisms were re-suspended in RPMI 1640 (Sigma-Aldrich, St. Louis, MO, USA). Both *Candida* and *Staphylococcus* suspensions were spectrophotometrically standardized at an OD540 nm of 1.0 and OD600nm of 0.1, respectively, which corresponds to a final concentration of 10^7^ cells per mL^-1^ [14].

### Adhesion and biofilm formation

Single species adhesion and biofilms assays were carried out on 96-well microplates (Orange Scientific, Belgium) containing 75 μL of RPMI 1640 and 75 μL of each cellular suspension. Dual species adhesion and biofilm formation were also performed in 96-well microplates containing 75 μL of the suspension of each microorganism used in the following associations: *C*. *albicans* + MSSA and *C*. *albicans* + MRSA. For the adhesion assays, culture plates were incubated at 37°C in an orbital shaker (75 rpm) for 90 min. For the biofilm formation, the plates were also incubated for 90 min at 37°C in an orbital shaker (75 rpm). The non-adherent cells were then removed by gently washing twice with 150 μL PBS and 150 μL of fresh RPMI 1640, added to each well. The plates were incubated for 12 h, 24 h, and 48 h. In the case of the 48 h biofilms, 75 μL of the suspensions were removed and an equal volume of fresh RPMI 1640 was added after the first 24 h.

### Quantification of adhered and biofilm cells

The number of adhered cells and those present in the biofilms was determined by counting colony-forming units (CFUs). The wells were briefly washed twice with PBS to remove loosely attached cells and resuspended in 100 μL of PBS. Next, the adhered biofilm was carefully scraped off the wells with a sterile pipette tip for 1 min [35]. Cristal violet (CV) staining as described below was used to confirm the complete removal of the adhered cells and biofilms. The 100 μL suspensions were then vigorously vortexed to separate a possible aggregation among the cells. Serial decimal dilutions (in PBS) were made and the number of *C*. *albicans* was determined by pipeting replicate specimens (25 μL) of the suspensions on SDA medium supplemented with chloramphenicol. The same procedures were performed for the MSSA/MRSA cells, which were plated on Mannitol Salt Agar [10,33]. For the dual species studies, serial dilutions were plated onto both media. The plates were incubated for 24h-48 h at 37°C and the values of CFU mL^-1^ were counted. The experiments were performed in five replicates and repeated in three independent assays.

### Quantification of metabolic mitochondrial activity

The total metabolic activity of adherent and biofilm cells was measured through the 2,3-bis reduction assay (2-methoxy-4-nitro-5-sulfophenyl)-5-[(phenylamino) carbonyl]-2H-tetrazoliumhydroxide (XTT) using a previous described method [36]. After 90 min of adhesion and 12 h, 24 h, and 48 h of biofilm formation, the XTT (Sigma) was prepared in ultrapure water at a final concentration of 1 mg mL^-1^. The solution was filter sterilized and stored at -70°C until use. The menadione solution (Sigma) was prepared in acetone at 0.4 mM immediately before each assay. The wells of a 96-well tissue plate were washed with 150 μL of PBS (0.1 M, pH 7) and a mixture of 158 μL YNB with 200 mM glucose, 40 μL XTT, and 2 μL menadione was added to each well. The plates were incubated at 37°C for 3 h in the dark and the absorbance at 492 nm was then read in an ELISA plate reader in the 96-well tissue plates. The experiments were performed in five replicates and repeated in three independent assays.

### Quantification of total biofilm biomass

Total biofilm biomass was measured using a CV staining method as described before [36]. Briefly, after 12h, 24h, and 48 h of biofilm formation, the medium was totally aspirated and the non-adherent cells removed by washing the wells with 150 μL of PBS. The single and dual-species biofilms were then fixed with 200 μL of 100% (v/v) methanol, which was removed after 15 min. The culture plates were allowed to dry at room temperature and 200 μL of CV (1% v/v) was then added to each well and incubated for 5 min. The wells were gently washed twice with 150 μL of PBS and 200 μL of acetic acid (33% v/v) was added to release and dissolve the stain. Optical density (Absorbance) of the eluted solution was read in a microtiter plate reader (Bio-Tek Synergy HT, Izasa) at 570 nm. The experiments were performed in five replicates and repeated in three independent assays.

### Quantification of extracellular hydrolytic enzymes

The supernatants of the 48 h single- and dual-species biofilms were centrifuged for 5 min at 5,000 rpm to remove any cells and evaluated for secreted aspartyl proteinase (SAP) activity using the fluorimetric EnzChek Protease Assay kit for green fluorescence (Molecular Probe, Inc., Eugene, OR, USA), according to the manufacturer's recommendations. Briefly, 0.2 mL of the BODIPY FL casein stock solution (1.0 mg mL^-1^) and 19.8 mL of the digestion buffer (diluted 1:20) were prepared and mixed. Thereafter, 100 μL of the working solution were added to 100 μL of the centrifuged supernatants in black 96-well microplates for fluorescence detection (Greiner Bio ONE, Frickenhausen, Germany) and the plates were incubated for 2 h at room temperature, protected from light. The fluorescence was read in a fluorescence microplate reader (Fluoroskan Ascent microplate fluorometer, USA) at 485 nm of excitation and 538 nm of emission. The fluorescence values were used in linear equations derived from the previously obtained standard curves and the enzymatic activity was expressed in μg mL^-1^.

For the phospholipase C (PL-C) assay, the 48 h single- and dual-species biofilms were resuspended in a lysis buffer (2 M Tris- HCI, 1 M CaCl_2_, ultra-purified water, pH 7.4), sonicated for 20 seconds to disrupt the biofilms, and centrifuged for 5 min at 10,000 rpm. To evaluate the PL-C activity, the Amplex Red phosphatidylcholine-specific phospholipase C assay kit (Molecular Probe, Inc., Eugene, OR, USA) was used, according to the manufacturer's recommendations. Briefly, a working solution was prepared mixing 200 μL of Amplex Red reagent stock solution (20 mM), 100 μL of horseradish peroxidase stock solution, 200 μL of alkaline phosphatase stock solution, 100 μL of choline oxidase stock solution, and 78 μL of the lecithin solution to 9.32 mL of Reaction Buffer (1:5). The assays were performed in black 96-well microplates, in a total volume of 200 μL per well (100 μL of the product of the centrifugation of the biofilms and 100 μL of the working solution). The plates were then incubated for 3 h at 37°C, protected from light. The fluorescence was read in a fluorescence microplate reader at 544 nm of excitation and 590 nm of emission. The fluorescence values (nm) were recorded and used for comparisons in relation to the fluorescence values of the positive controls provided by the manufacturer [purified PL-C from *Bacillus cereus* and Hydrogen Peroxide (H_2_O_2_)].

For both enzymes, all tests were performed on three separate occasions, with four samples in triplicate for each experimental condition.

### Scanning electron microscopy

Scanning electron microscopy (SEM) was used to examine the biofilm structures and the interactions between the *Candida* and *Staphylococcus* cells. For this assay, single- and dual-species biofilms were formed on polystyrene disks cut to loosely fit in the 24-well microplates (Orange Scientific, Belgium). Thus, after 90 min, 12h, 24h, and 48 h of incubation, the medium was aspirated and the non-adherent cells removed by washing twice with 1 mL of PBS. The discs were then dehydrated with an ethanol series (70% ethanol for 10 min, 95% ethanol for 10 min and 100% ethanol for 20 min) and air dried for 20 min. Disc samples were kept in a desiccator before analysis. Prior to observation, the discs were mounted onto aluminum stubs sputter coated with gold and observed using a Hitachi Tablelap Scanning Electron Microscope TM-3000 (Tokyo, Japan).

### Statistical analysis

The results of the CFU, CV, and XTT reduction values were compared using two-way ANOVA with the Tukey post-hoc test. The two factors considered were growth condition (*C*. *albicans*, MSSA, MRSA, *C*. *albicans* + MSSA, and *C*. *albicans* + MRSA) and time period (90 min, 12h, 24h, and 48h for the CFU mL^-1^ and XTT assays, and 12h, 24h, and 48h for the CV assay).

For the SAP and PL-C activities, only the growth factor condition was analyzed using one-way ANOVA (Welch ANOVA in cases of unequal variance) followed by the Games-Howell post hoc test.

All tests were performed using the SPSS statistical software package (SPSS Inc., Chicago, USA) with a confidential level of 95%.

## Results

### Quantification of adhered and biofilm cells

**Fig 1:**
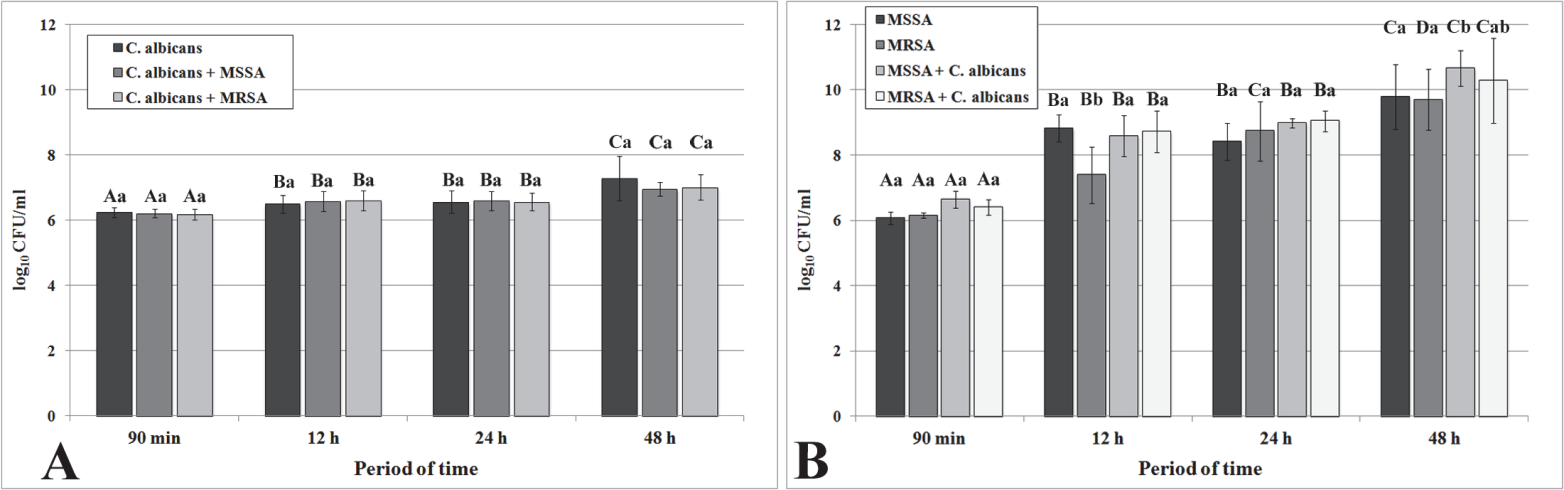
Mean log_10_ CFU mL^-1^ values for grown of *C*. *albicans* (A), MSSA and MRSA (B) as single- and dual-species grown in RPMI at 37°C. Error bars represent standard deviation. The uppercase letters show differences among time periods and lowercase letters show differences among the growth conditions (Tukey post-hoc test at ρ>0.05).

Table 1 shows the summary of the ANOVAs for all virulence factors analyzed. *C*. *albicans*, MSSA and MRSA were able to adhere (90 min) and to form biofilms either in single state or in mixed cultures. Table 1 shows that the time period factor had a significant effect (p<0.0001) on the log_10_ CFU mL^-1^ values of *C*. *albicans*. Fig 1A shows a small but gradual increase in the log_10_ CFU mL^-1^ values over time and, at 48 h, the number of cells of all growth conditions was significantly higher than in the other time periods (ρ<0.05).

**Table 1:** ANOVAs for mean values of log_10_ CFU mL^-1^, Abs CV, Abs XTT, SAP activity, and PL-C activity for *C*. *albicans* and bacteria results.

			DF^†^	F-value	ρ-value
**log**_**10**_ **CFU mL** ^-1^	***C*. *albicans***	**Period of time**	3	56.5166	<0.0001*
		**Growth condition**	2	0.7846	0.5381
		**Interaction**	6	1.5518	0.1635
	**Bacteria**	**Period of time**	3	337.5055	<0.0001*
	**(MSSA/MRSA)**	**Growth condition**	3	15.1278	<0.0001*
		**Interaction**	9	4.1278	0.0002*
**Abs CV**		**Period of time**	2	44.7022	<0.0001*
		**Growth condition**	4	206.437	<0.0001*
		**Interaction**	8	3.7525	0.0006*
**Abs XTT**		**Period of time**	3	268.3263	<0.0001*
		**Growth condition**	4	187.3755	<0.0001*
		**Interaction**	12	21.2554	<0.0001*
**SAP activity**		**Growth condition**	3	326.653	<0.0001*
**PL-C activity**		**Growth condition**	6	82.697	<0.0001*

^†^DF: Degrees of Freedom.

*Significant differences at ρ<0.05.

For the bacteria, both factors analyzed had a significant effect (ρ<0.0001) on the log_10_ CFU mL^-1^ values (Table 1). Regardless of whether they were grown as single- or dual-species biofilms, there was a gradual increase in the log_10_ CFU mL^-1^ values over time (Fig 1B), reaching the peak values at 48 h (ρ<0.05). It was also observed that the number of MSSA cells increased significantly (ρ<0.05) when cultured in the presence of *C*. *albicans* at 48 h (Fig 1B).

### Quantification of metabolic mitochondrial activity

**Fig 2:**
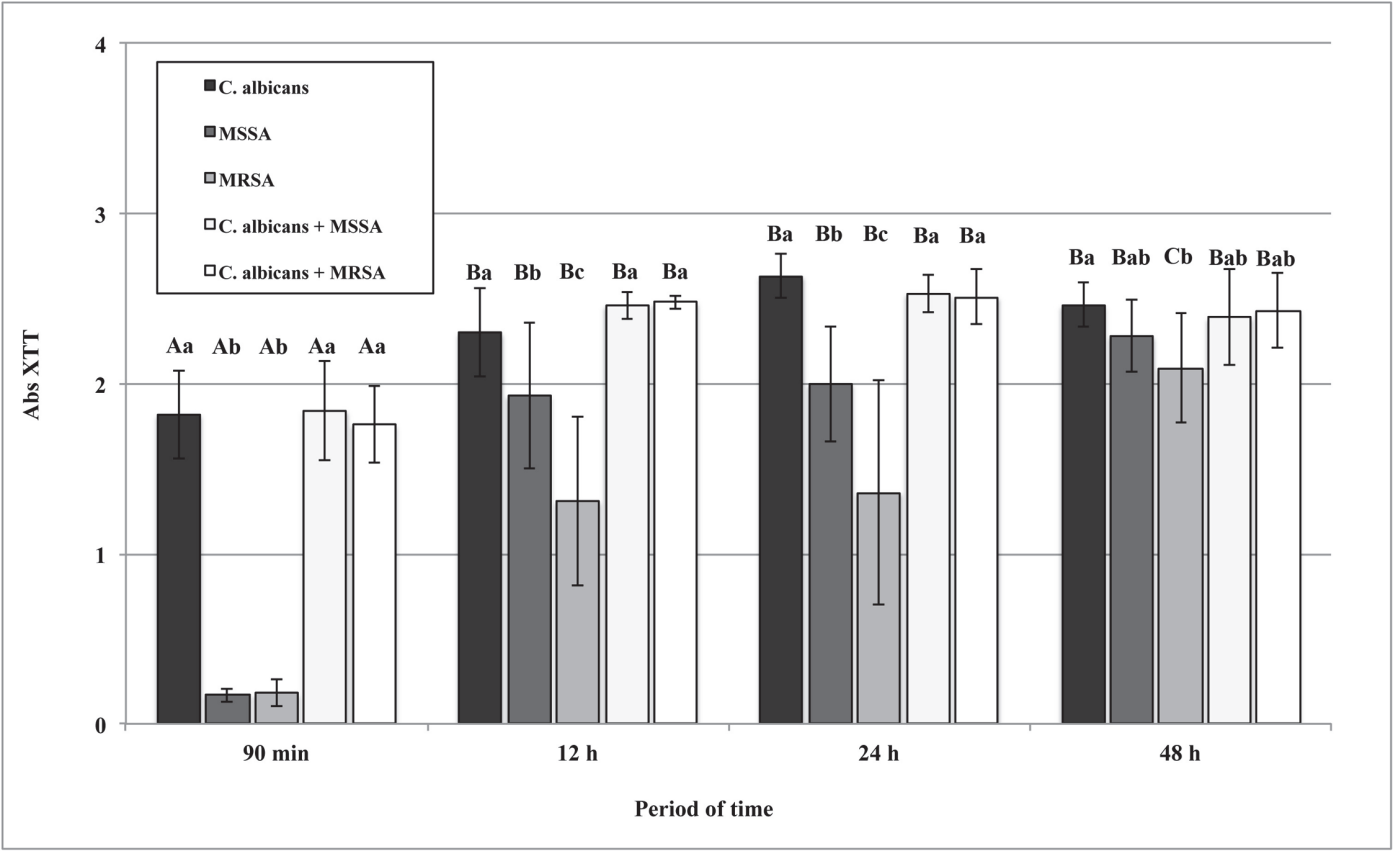
Absorbance values of XTT reduction (Abs XTT) obtained from 90 min of adhesion and biofilms of *C*. *albicans*, MSSA, and MRSA as single- and dual-species grown in RPMI at 37°C. Error bars represent standard deviation. The uppercase letters show differences among time periods and lowercase letters show differences among the growth conditions (Tukey post-hoc test at ρ>0.05).

Two-way ANOVA revealed significant effects for the time period and growth condition for the Abs XTT values (Table 1). Except for the MRSA single biofilms, that showed higher metabolic mitochondrial activity at 48 h, all microorganisms in both growth conditions had a significant increase (ρ<0.05) in the biofilm metabolic activity from the adhesion period to 12 h of biofilm formation, after which no changes were observed (Fig 2).

When the growth condition factor was analyzed, from the adhesion phase to the 24 h biofilm formation, there were no significant differences (ρ>0.05) in the metabolic activity between the single and dual cultures with *C*. *albicans*, which had higher values of Abs XTT (ρ<0.05) than the single bacteria cultures. At 12 h and 24 h of biofilm growth, the MRSA single-species biofilm presented significantly lower values (ρ<0.05) of metabolic activity than the MSSA. At 48 h of biofilm formation, there were no significant differences (ρ>0.05) among single- and dual-species biofilms, with the exception of the *C*. *albicans* biofilm, which presented higher metabolic activity than that of the MRSA (ρ<0.05).

### Quantification of total biofilm biomass

**Fig 3:**
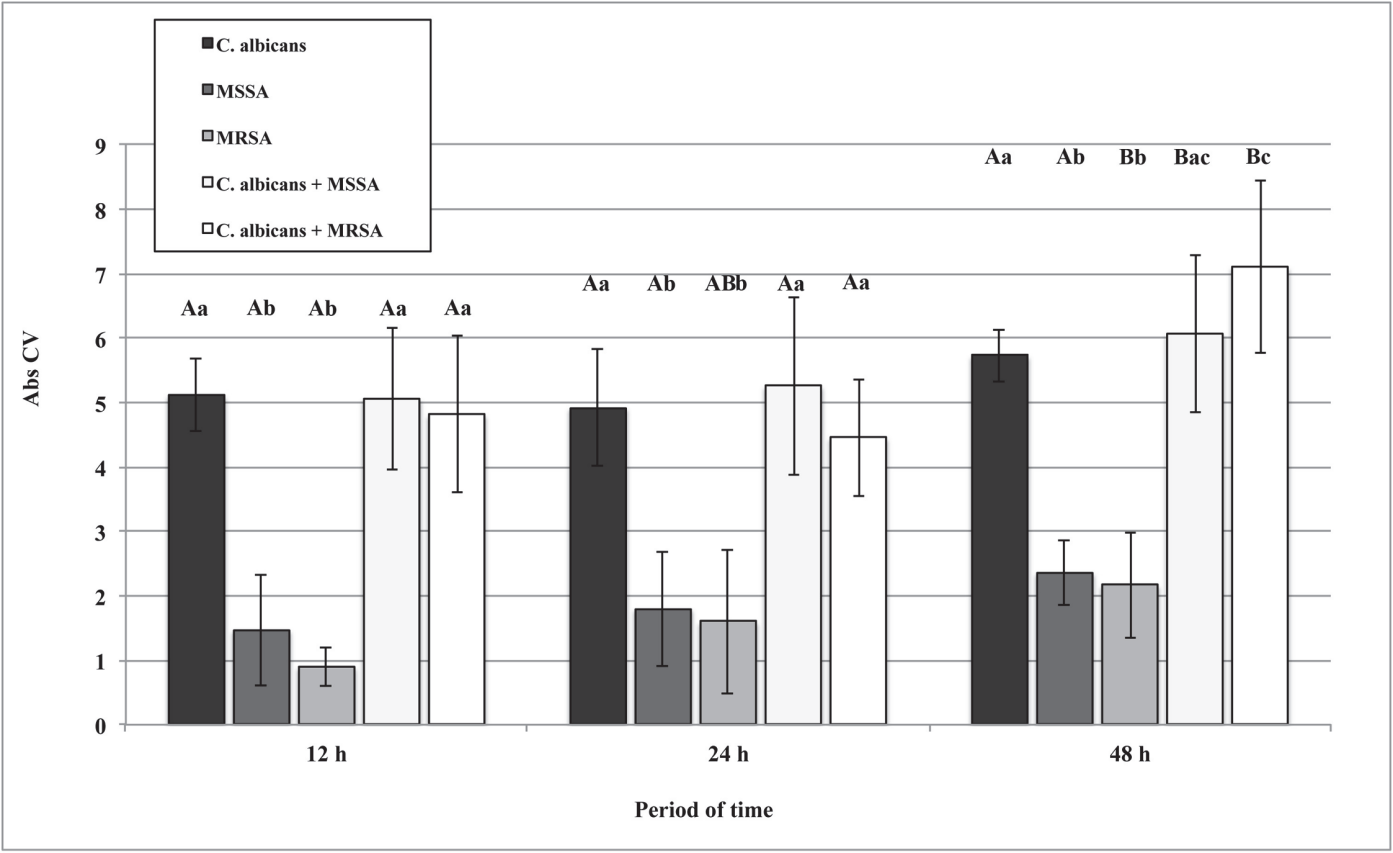
Absorbance values of CV solutions (Abs CV) obtained from biofilms of *C*. *albicans*, MSSA, and MRSA as single- and dual-species grown in RPMI at 37°C. Error bars represent standard deviation. The uppercase letters show differences among time periods and lowercase letters show differences among the growth conditions (Tukey post-hoc test at ρ>0.05).

Two-way ANOVA revealed significant effects for the time period and growth conditions for the Abs CV values (Table 1). Fig 3 presents the results for total biomass of single and dual biofilms of *C*. *albicans*, MSSA and MRSA. Generally, *C*. *albicans* single and mixed biofilms had significantly higher total biomass values (ρ<0.05) than the single biofilms of bacteria. Except for the significant increase in total biomass of the single MRSA biofilm at 24 h, all growth conditions showed no changes from 12 h to 24 h biofilm formation (ρ>0.05). While both mixed biofilms increased the total biomass from 24 h to 48 h (ρ<0.05), no significant change was observed for the *C*. *albicans* single biofilm.

### Quantification of extracellular hydrolytic enzymes

**Fig 4:**
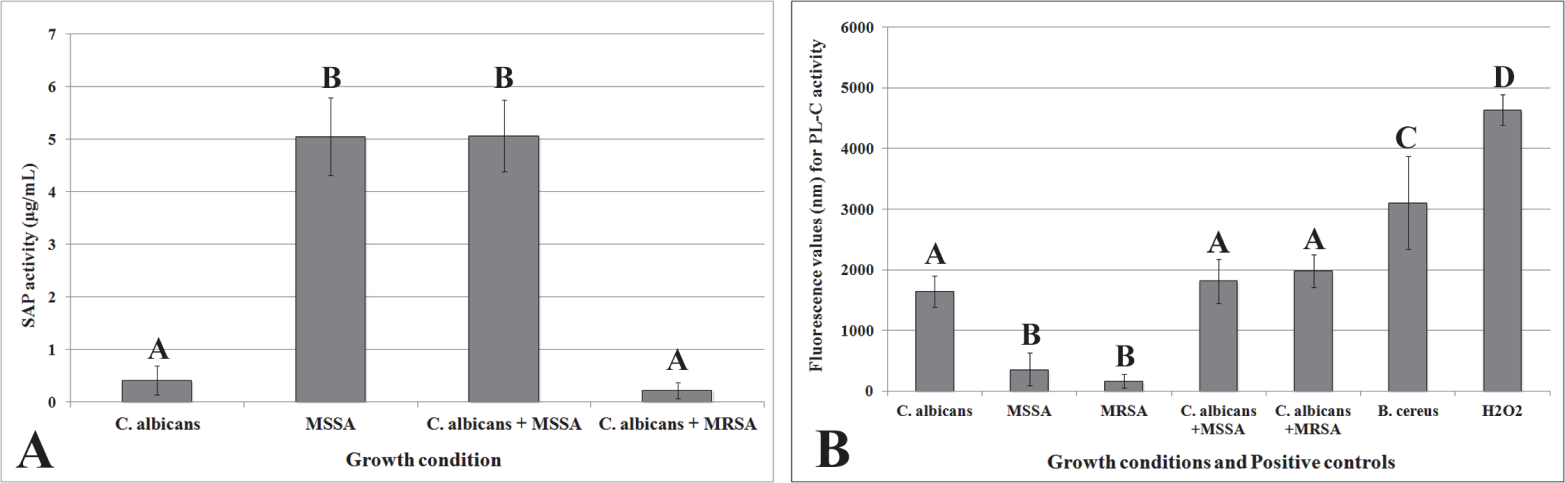
SAP (A) and PL-C (B) activities of biofilms of *C*. *albicans*, MSSA, and MRSA, as single- and dual-species grown in RPMI at 37°C, and the positive controls. Error bars represent standard deviation. The uppercase letters show differences among the growth conditions (Games-Howell post-hoc test at ρ>0.05).

One-way Welch ANOVA revealed significant effects of growth condition in the SAP and PL-C activities (Table 1). For the SAP activity, the single biofilm of *C*. *albicans* and the mixed biofilm of *C*. *albicans* plus the MRSA produced significant lower amounts (ρ<0.001) of SAP when compared to the single biofilm of MSSA and the mixed biofilm of *C*. *albicans* plus MSSA (Fig 4A). The enzymatic activity of the single biofilm of MRSA was not detected using the fluorimetric kit. The fluorimetric values for PL-C activity are presented in Fig 4B. *C*. *albicans* single and mixed biofilms had significantly higher fluorimetric values (ρ<0.05) than the single biofilms of bacteria. Both positive controls showed higher fluorimetric values (ρ<0.05) than *C*. *albicans*, MSSA, and MRSA in all growth conditions.

### Scanning electron microscopy

**Fig 5:**
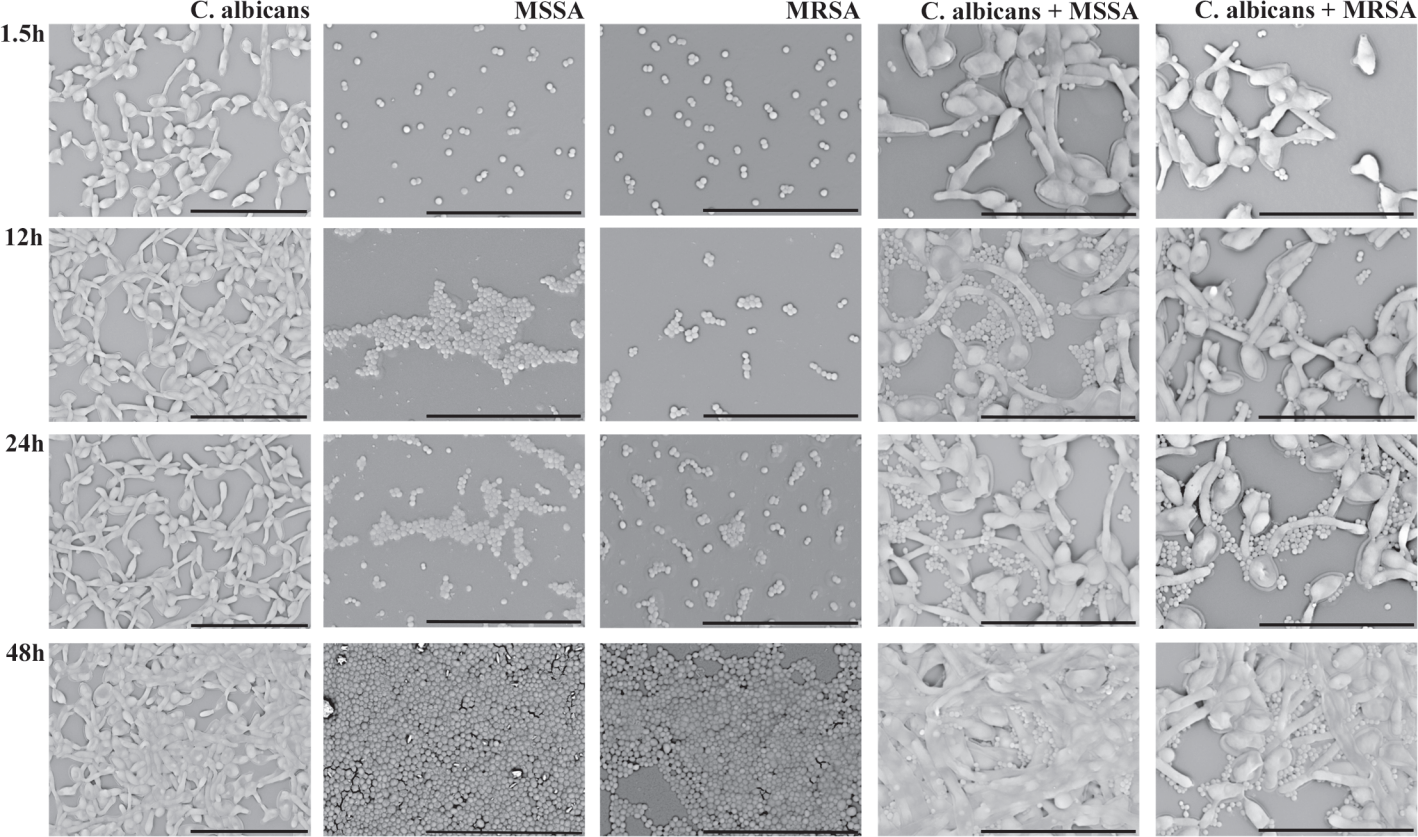
Scanning electron microscopy of adhered single- and dual-species (90 min) and biofilms (12 h, 24 h, and 48 h) of *C*. *albicans*, MSSA, and MRSA in RPMI at 37°C. The bar in images corresponds to 20 μm for *C*. *albicans* (magnification of x2500) and 20 μm for bacteria and mixed growth (magnification of x5000).

Scanning electron microscopy was used to examine the single and dual adhered and biofilm species in terms of structure and interactions between the candidal and bacteria cells (Fig 5).

The single *C*. *albicans* showed, at 90 min, a non-contiguous layer of cells, which presented an initial formation of hyphal forms, and evolved into mature biofilm producing a thickness of co-aggregated cells with a higher number of hyphae at 12 h, 24 h, and 48 h. It is important to emphasize that the 48 h biofilm formation resulted in a more robust biofilm (Fig 5). The single MSSA and MRSA, at the initial phase of 90 min, revealed a similar extent of adhesion with lower quantity of uniformly distributed cells. The structure of both biofilm strains was comparable, despite that the MSSA biofilms presented a higher number of intimately packed cocci (Fig 5).

Fig 5 also represents the dual species physical interactions. Images revealed extensive adherence of MSSA and MRSA to *C*. *albicans*, with a preferential association to the hyphal elements of *C*. *albicans* and to a lesser degree, the round yeast cells. In fact, after 90 min of interaction, the bacteria cells were able to interact with the *C*. *albicans* cells. In addition, it is important to highlight that in areas of dense hyphal biofilm growth, the MSSA and MRSA cells appear in higher quantity than in the well surface.

## Discussion

In the present study, the CFU, XTT, and crystal violet assays were used to evaluate the interactions of single and dual biofilms because these methodologies are complementary. The crystal violet biomass assay quantifies the matrix of both living and dead cells. Because all cells and matrix are stained in this method, it provides the general condition of the biofilm. However, neither the viable cells of the biofilm nor their metabolic activity is evaluated. To discriminate between living and dead cells, the CFU method was chosen because it is the widely gold standard method used. Its noteworthy advantage is that only the viable cells are counted, excluding the dead ones and debris. However, this method quantifies all viable cells, including those with low metabolic activity. Thus, another quantification method based on the metabolic activity (XTT) was used in the present study. For all methodologies, the biofilm formation was stopped at 48 h because the biofilms are sufficiently mature at this time point [2,37,38]. In addition, this protocol of biofilm formation has already been adopted by other studies that evaluated single and dual biofilms [9,13].

The results of the present investigation showed that all three pathogenic microorganisms were able to adhere in a similar extent, with no apparent antagonism (Fig 1). This initial attachment of cells was followed by cell division, proliferation, and biofilm development. The current study also demonstrated that, under the conditions tested, all strains were able to form biofilms, although in different extents (Figs 1, 2, 3, and 5). Indeed, it was not observed that the bacteria’s number decreased over time in the presence of *C*. *albicans*, indicating the ability of the yeasts and bacteria to co-inhabit. *C*. *albicans* levels in single- and dual-biofilms were not different (Fig 1 A). It is important to emphasize that, in the presence of *C*. *albicans*, the number of bacteria cells increased in specific periods of time comparatively to its single biofilms (Fig 1 B). Similar results were also observed by Harriott & Noverr [5], who monitored fungal and bacterial growth within biofilms and observed that while the *C*. *albicans* levels presented no changes in the single- and dual-biofilms, the *S*. *aureus* levels were significantly increased in the dual biofilms. A possible explanation for this phenomenon could be the presence of higher quantity of glucose, a major component of the *C*. *albicans* matrix [39]. In fact, glucose can provide a carbon source for *S*. *aureus* growth and increase its biofilm formation [5].

Considering the metabolic activity assay, it was observed that, from the adhesion phase until the earlier biofilm formation at 24 h, the metabolic activity of single and dual cultures with *C*. *albicans* were higher than the single bacteria, as expected. In fact, single MSSA and MRSA biofilms only achieved similar metabolic activity values at the 48 h period. Since biofilm cells are organized into structured communities embedded within an extracellular matrix, activity within biofilms would be dependent on nutrient access and availability of oxygen, together with removal of waste products [35]. These factors may vary because of inherent differences in the biofilms produced by the different microorganisms tested here, resulting in variations in biofilm metabolic activity. In addition, in the present study, RPMI media was used for biofilm formation because, according to the literature [40], it induces hyphal development. This may have influenced the metabolic results due to the lack of some nutrients for both microorganisms. From Fig 3, it can be seen that both the *C*. *albicans* and dual species biofilms produced higher biofilm biomass than the bacteria monomicrobial biofilms. Bamford et al. [41], who investigated a biofilm formed by *Streptococcus gordonii* and *C*. *albicans*, also noted that complex physical and chemical interactions between microorganisms resulted in an increased biomass of co-inoculated biofilms. More recently, a similar interaction pattern has been reported for *C*. *albicans* and MRSA [42].

It is important to highlight that a direct comparison of total biofilm biomass and metabolic activity may mislead, since there are significant differences between bacteria and yeasts, including relative cell size, morphology, and biochemistry. Despite the fact that the literature is sparse on the effects of microbial interaction on metabolic activity, our results are corroborated by a recent study, in which dual biofilms of *C*. *albicans* and *S*. *aureus* showed higher metabolic activity than the single bacteria biofilms [13]. This result is important given the fact that biofilm activity is significant in terms of pathogenicity since it is a likely indicator of growth, production of hydrolytic enzymes and, in the case of reduced activity, of possible resistance to antimicrobial activity [35]. The ability of these two organisms to maintain the growth and metabolic activity when they are together may have potential clinical implications for polymicrobial infections. In fact, *in vivo* studies demonstrated that mice inoculated with *S*. *aureus* showed low mortality, whereas the co-inoculation with *C*. *albicans* resulted in a higher rate of mortality [11,42]. A recent study also found that co-infection with these microorganisms led to a 40% mortality rate and increased microbial burden in the spleen and kidney by day 1 post-infection [42]. Thus, the interaction between these two organisms appears to be beneficial to each other.

According to the authors’ knowledge, there is a lack of studies evaluating the secretion of degradative enzymes by dual biofilms of *C*. *albicans* and *S*. *aureus*. When compared to other virulence attributes, the secretion of SAP and PL-C has a more pronounced influence on the pathogenicity of the microorganisms. Lyon *et al*. [43] found a positive correlation between higher SAP and PL-C production and greater adhesion to buccal cells and resistance to fluconazole of *C*. *albicans* isolates. In addition, there is evidence showing that biofilm formation is also positively associated with secretion of hydrolytic enzymes [44]. In the present study, for single biofilms, *S*. *aureus* showed higher SAP activity when compared to *C*. *albicans*, which in turn produced higher PL-C activity. Interestingly, when the microorganisms were in a co-culture, both enzymes were also produced (Fig 4), which suggests an increased pathogenicity of dual biofilms. Kaminishi and colleagues [45] reported that the SAP produced by *C*. *albicans* appears to reduce opsonization and phagocytosis of *S*. *aureus* by polymorphonuclear leukocytes, so that the breakdown of the humoral host defense mechanisms caused by the action of *C*. *albicans* SAP can leave the hosts more vulnerable to microbial infections and aggravate infectious diseases in compromised hosts. In addition, the secretion of SAP by *Staphylococcus* could help *C*. *albicans* to enhance its adhesion to the mucosal layer [46]. The specific roles of these two hydrolytic enzymes in colonization and infection processes are slightly different. In general, SAPs induce the degradation of a large variety of host proteins, increasing the ability of microorganisms to colonize and invade host tissues [47]. SAPs are also able to break down portions of immunoglobulins, which are glycoproteins synthesized and secreted by plasma cells that function as antibodies. This mechanism plays a major role in disrupting the humoral host defense [45,48]. The extracellular PLs are capable to target and digest the membrane phospholipids, the major component of all host cell membranes, thereby facilitating microorganism invasion [49]. Since PLs are concentrated mostly in the tips of the hyphae, such host cell injury would be expected to facilitate the penetration of the infecting microorganisms [50]. In addition, PLs have been found to be a potent inflammatory agent, inducing the accumulation of inflammatory cells and plasma proteins and release of various inflammatory mediators *in vivo* [50]. The fluorimetry methods used in the present study were not able to detect the secretion of SAP by the single biofilm of MRSA. This method is considered more sensitive than the colorimetric’s [27,51–53] and those based on the formation of an opaque degradation halo in specific agar plates [28,54–57]. In addition, the fluorimetry assays are considered suitable for research involving biofilms [53] and strains with low levels of enzymatic activity [50,56].

The present investigation also showed that extensive interactions did occur between *C*. *albicans* and the bacteria growing in mixed biofilms and, in general, *C*. *albicans* favors bacterial growth. SEM images revealed an extensive adherence of MSSA and MRSA to *C*. *albicans*, with a preferential association to the invasive hyphal elements of *C*. *albicans*. In areas of dense hyphal biofilm growth, the *S*. *aureus* cells could be seen completely covering the *C*. *albicans* hyphae (Fig 5). Peters et al. [14] also observed an association between *S*. *aureus* cells and invasive hyphal forms of *C*. *albicans*, but not the round yeast cells. This interaction was further elucidated by these authors, who observed that *S*. *aureus* binds to *C*. *albicans* agglutinin-like sequence 3 (*als3*), which plays a key role in mediating the adherence of bacteria cells to *C*. *albicans* hyphae [23]. Because *S*. *aureus* preferentially associates with the hyphae, Harriott and Noverr [12] suggested that dual biofilm formation is initiated after *C*. *albicans* adheres to a substrate surface, which initiates germination. Attachment of *S*. *aureus* to the germinating hyphae would presumably occur at the substrate surface and continued growth of both species occurs concomitantly. This observation is supported by previous studies that showed that *S*. *aureus* was found throughout the depth of the dual biofilm with microcolonies on the surface [5,12,23]. Moreover, it has been demonstrated that, when *S*. *aureus* was associated with the hyphal forms, it was surrounded with a distinct halo of matrix material secreted by *C*. *albicans*. As a result, *S*. *aureus* resistance to vancomycin was increased, probably due to the matrix that may limit penetration of the drug into the biofilm [5].

Mono or polymicrobial infections are complex processes regulated by a dynamic equilibrium among the host immune defense, microorganisms’ interaction and their virulence factors. This study demonstrated that *C*. *albicans*, MSSA, and MRSA can co-exist in a biofilm mode of growth, in an apparent synergistic effect, with bacteria cells preferentially associated to hyphal forms. In general, the dual biofilms showed an evolutive behavior over time in terms of number of cells, metabolic activity, and total biomass. Furthermore, when *C*. *albicans*, MSSA, and MRSA were in a co-culture, both SAP and PL-C enzymes were produced, which may suggest an increased severity of dual biofilms. Although it is unlikely that the specific resistance mechanisms to antibiotics of MRSA (presence of mecA gene) would influence the *Candida*-bacteria interactions, it is important to highlight that other factors determining the difference between MRSA and MSSA strains must exist, and should be further investigated, excluding differences in drug resistance mechanisms. In fact, our results showed some significant differences between bacteria biofilms and their combination with *C*. *albicans*. This indicates that, despite both bacterial populations are virulent, the mechanisms of pathogenesis of MRSA and MSSA infections may differ at some degree. With that in mind, this study contributed to the understanding of the complex interactions between microbial species, which would provide a new perspective for developing new strategies for the prevention, control and treatment of polymicrobial infections. The identification of potential targets for the inhibition of co-adhesion, biofilm development, and enzymes secretion may ultimately provide the means to modify microbial colonization and infection, reducing the impact of polymicrobial diseases on human health.
